# Comment on ‘Prevalence of depression in patients with sarcopenia and correlation between the two diseases: systematic review and meta‐analysis’

**DOI:** 10.1002/jcsm.13027

**Published:** 2022-06-17

**Authors:** Xiao‐Ming Zhang, Xinjuan Wu

**Affiliations:** ^1^ Department of Nursing, Chinese Academy of Medical Sciences‐Peking Union Medical College Peking Union Medical College Hospital (Dongdan campus) Beijing China

With great interest, we have read a recent systematic review and meta‐analysis conducted by Li *et al*.^1^ who summarized the prevalence of depression among sarcopenic people and pooled the association between sarcopenia and depression. They did a great job for identifying the evidence of depression among sarcopenic people and providing more information about the importance of sarcopenia. Furthermore, the authors did a broad subgroup analysis, which helped us to understand the association between sarcopenia and depression in different variables. However, there are still some issues worth discussing and addressing.

First, we believe two studies published before 31 August 2021 that met the inclusion and exclusion criteria should be included in this systematic review and meta‐analysis. The first study was reported by Chen *et al*.^2^ who conducted the prevalence of sarcopenia and its risk factor among older adults from the Chinese community. One hundred and seventy‐two older adults had sarcopenia, and the prevalence of depression assessed by the Geriatric Depression Scale was 9.8%. In addition, the authors declared that cross‐sectional and cohort study design was one of the inclusion criteria. However, a cohort study conducted by Chen *et al*.^3^ was not in this systematic review. In this cohort study, Chen *et al*.^3^ found that the baseline sarcopenia was an independent risk for 1‐year depression after adjusting potential confounding factors with the odds ratio (OR) being 3.57 [95% confidence interval (CI):1.59–8.04]. We have pooled the prevalence of depression among sarcopenic people, and the results showed that the pooled prevalence of depression was 27% (19–35%), which was slightly lower than Li's study [28% (21–36%)], as shown in *Table* 1.

**Table 1:** Summarized the results of meta‐analysis and publication bias

Meta‐analysis	Numbers of studies	Prevalence/OR	95%CI	*I* ^2^	*P* value for publication bias^a^
Prevalence of depression	16	27%	19−35%	76.4%	*P* = 0.017
The association between sarcopenia and depression	17	2.00	1.55−2.57	54.19%	*P* = 0.016

CI, confidence interval; OR, odds ratio.

^a^ Begg's test for publication bias.

Second, we pooled the association between sarcopenia and depression by adding the cohort study, and we found that the pooled OR was 1.80 (95%CI: 1.41, 2.30). However, there was high heterogeneity across these included studies; therefore, it is inappropriate to pool these studies. Then, we used sensitivity analysis to detect which studies were the main reason for this high heterogeneity, and the results found two studies (Endo *et al*.^4^ and Fábrega‐Cuadros *et al*.^5^) that made a remarkable change for the overall effect size when added in or eliminated that. Based on the sensitivity analysis, we believe the abovementioned studies were one of the main possible reasons for the heterogeneity, which should be adopted as a single descriptive analysis. We pooled the other studies and found a positive association between sarcopenia and depression among people, with the pooled OR being 2.00 (95%CI: 1.55, 2.57), as shown in *Table* 1. The heterogeneity across these studies had a remarkable decrease, changing from 76.4% to 54.19%.

Thirdly, we believe the authors should perform publication bias in this meta‐analysis. The publication bias test by Begg found potential publication bias in this systematic review and meta‐analysis (*P* < 0.05) (*Table* 1). Some reasons might explain these results. First, this meta‐analysis only included English‐language studies in a few databases; other Chinese internet databases such as WanFang and CNKI might provide important studies. Second, studies with negative results might did not have an appropriate opportunity for publication.
